# Analyses of Progressive Supranuclear Palsy Quality of Life in Frontotemporal Lobar Degeneration: Findings from the ALLFTD Consortium

**DOI:** 10.1002/alz.092005

**Published:** 2025-01-09

**Authors:** Kevin M Nelson, Jeremy A. Syrjanen, Leah K. Forsberg, Hilary W. Heuer, Adam L. Boxer, Howard J. Rosen, Brad F. Boeve

**Affiliations:** ^1^ Mayo Clinic, Rochester, MN USA; ^2^ University of California, San Francisco, San Francisco, CA USA; ^3^ University of California San Francisco, San Francisco, CA USA

## Abstract

**Background:**

The Progressive Supranuclear Palsy Quality of Life (PSP‐QoL) scale is a 45‐question participant‐completed questionnaire used to evaluate physical and cognitive task difficulties. Participants enrolled in the ALLFTD research program are asked to complete this questionnaire annually during their study visits.

**Method:**

PSP‐QoL responses were analyzed at baseline visits. Our analysis focused on responses to the overall satisfaction of life (range 0‐100, with 0 being extremely dissatisfied and 100 being extremely satisfied) relative to disease severity (CDR®+NACC‐FTLD global scores) and as a function of clinical phenotypes. A Kruskal‐Wallis test was used to compare overall satisfaction ratings against CDR®+NACC‐FTLD global score.

**Result:**

788 PSP‐QoL survey responses were analyzed (participant mean age 55.3 ±15 years, 50.5% female). Analysis included responses from 372 (47.2%) asymptomatic participants (CN; CDR®+NACC‐FTLD = 0), 140 prodromal (17.8%) CDR®+NACC‐FTLD = 0.5, 160 (20.3%) CDR®+NACC‐FTLD = 1, 106 (13.5%) CDR®+NACC‐FTLD 2, and 10 (1.3%) CDR®+NACC‐FTLD = 3. All symptomatic groups were similar in PSP‐QOL overall satisfaction of life rating: (mean overall satisfaction CDR®+NACC‐FTLD = 0.5: 74.5 (SD 22.5); CDR®+NACC‐FTLD = 1: 74.1 (SD 24.7) and CDR®+NACC‐FTLD = 2/3: 74.1 (SD 25.8)), but these differed from asymptomatic (cognitively normal): (mean 83.7, (SD 14.6); p<0.0001). Similarly, when including the CN group and symptomatic phenotype groups, a statistically significant difference in ratings was seen: mean overall satisfaction CN = 83.7 (SD 14.7), MCI = 78.3 (SD 16.6), PPA = 71.6 (SD 26.9), PSP = 66.3 (SD 28.2), bvFTD = 76.8 (SD 23.8), and other = 70.6 (SD 23.2). However, even after removing the CN group, a difference amongst symptomatic phenotypes remained (p = 0.036).

**Conclusion:**

Mean overall life satisfaction as measured by the PSP QoL questionnaire was lower in all symptomatic compared to clinically normal participants, regardless of disease severity or phenotype. Further longitudinal analyses are needed to better understand how clinical progression impacts overall satisfaction of life.

